# The role of substance use in the risk of not getting employed among young people: Prospective findings from the CONSTANCES cohort

**DOI:** 10.1192/j.eurpsy.2022.244

**Published:** 2022-09-01

**Authors:** R. El Haddad, J. Matta, C. Lemogne, M. Melchior, M. Zins, G. Airagnes

**Affiliations:** 1 Population-based Epidemiological Cohorts Unit, Inserm Ums 011, Villejuif, France; 2 INSERM UMS 011, Population-based Epidemiological Cohorts Unit, Villejuif, France; 3 AP-HP, Assistance Publique - Hôpitaux de Paris, Adult Psychiatry, Paris, France; 4 INSERM, Sorbonne University, Team Of Social Epidemiology (eres), Pierre Louis Institute Of Epidemiology And Public Health (iplesp), Paris, France; 5 Université de Paris, AP-HP, Hôpital Européen Georges Pompidou,, Dmu Psychiatrie Et Addictologie, Centre Ambulatoire D’addictologie, Inserm, Population-based Epidemiological Cohorts Unit, Ums 011,, Villejuif, France

**Keywords:** Cannabis use, Alcohol use, Tobacco use, Employment

## Abstract

**Introduction:**

It remains unclear whether substance use in youth could be associated with a lower likelihood of accessing employment.

**Objectives:**

To examine prospectively associations between substance use and the risk of not getting employed among young people.

**Methods:**

From the French population-based CONSTANCES cohort, 2,873 students who never worked were included between 2012 and 2018 and followed-up for 2.7 years in average. Generalized estimating equations computed the odds of being unemployed versus employed according to substance use at baseline controlling for sociodemographic factors and depressive state. Tobacco use (smoking status and number of cigarettes), cannabis use frequency, and at-risk alcohol use according to the Alcohol Use Disorder Identification Test (total score >7) were introduced separately in the models.

**Results:**

Tobacco use wasn’t significantly associated with employment. Cannabis use at least weekly, and at-risk alcohol use, were associated with increased odds of being unemployed (OR=1.85, 95%CI(1.29, 2.64)) and OR=1.34, 95%CI(1.04, 1.71)), respectively. Additional analyses on sub-scores of alcohol use suggested that the association was mainly driven by alcohol dependence rather than frequency of use.

**Conclusions:**

Public health campaigns must target youth by advising them of the detrimental roles of regular cannabis use and at-risk alcohol use and their lower chances of getting employed.

**Disclosure:**

No significant relationships.

